# Mr. Whitlam, on the Use of Conium Maculatum

**Published:** 1805-08-01

**Authors:** John Whitlam

**Affiliations:** Nottingham


### *To the Editors of the Medical and Physical Journal.*

Gentlemen,

AS the greatest dissimilarity of opinion exists amongst eminent professional characters, respecting the curative powers of several active medical agents; it might be of considerable advantage to practitioners in general, were the causes of this difference of opinion successfully investigated.

I flatter myself, that with regard to some of the remedies above alluded to, my researches have not been altogether without effect. Having frequently experienced the most beneficial results from the use of conium maculatum in preternatural enlargements of various parts of the human body; and as this herb has been as much recommended by some, as decried by others of equal rank and respectability as physicians, I make choice of it as an example.

Conium is an herb which requires not the chemist's art. Its virtues are concentrated in the expressed juice of its leaves, or by an infusion of the herb in boiling water. Thus prepared, it will be found capable of exerting all its energy; whether used internally, or externally applied. Whoever uses it in either of these forms, will never afterwards be inclined to trust to the extract of the plant; which is always divested of the volatile parts, on which its discutient property seems to depend. But the great source of error in regard to this vegetable, which has frequently quently deceived those who prescribed it, is the ignorance of the people employed to gather it. Those unacquainted with its botanical characters, frequently get the chaerophyllum instead of the conium. This has been remarked by the ingenious translator of the Edinburgh New Dispensatory. When this mistake takes place, it sufficiently accounts for the apparent want of efficacy in the plant. Let those therefore, who have despised this active remedy, only procure it genuine; let them use it in its simplest forms, and persevere a few weeks in its use; and then, if they find it inefficacious, let them report it to the medical world.

The following case, in which it produced the most astonishing effects, will serve as an additional proof of its great powers.

W. P. a person between forty and fifty years of age, formerly of an extremely robust habit, capable of bearing the greatest excesses and fatigues, had the venereal disease repeatedly. The symptoms were in general removed by drastic remedies; principally of his own prescribing. The last time, he caught the disorder in the form of gonorrhœa virulenta. He took calomel mixed with some purgative pill. The discharge from the urethra ceased, and has not since returned. In June last, it being several months after he was apparently cured, his throat became ulcerated, and his skin was partially covered with venereal blotches. He applied to me, and the above named symptoms were removed by mild mercurial medicines and the decoction of the woods. One of his testicles now began to swell, that enlargement was speedily removed by the ordinary means. He then had pains similar to those caused by acute rheumatism; these alternated with soreness of the throat and blotches on the skin. In October, the disease seemed nearly taken away. In January, his testicle began again to swell; and together with the spermatic cord, &c. it became more than four times its original size. The parts were extremely hard, and he had so much pain as to preclude the possibility of taking any repose, although opium was given in large doses. An eminent surgeon was called in. Discutient topical remedies were used, and mercurials and anodynes given internally. After a considerable time, no advantage was gained. Green hemlock was then with difficulty procured. A strong fomentation was made with the roots. It was applied several times a day; and after each fomentation, a poultice made of bread-crumbs, water, and the young shoots of the herb. In less than a week he passed passed comfortable nights. The swelling began to abate. The remedy was used six weeks. At this time the parts seem in their natural state, and no symptom of lues has appeared, unless we suppose the pain he has in his joints to arise from latent venereal virus. This may be the case; but if we judge from the symptoms, dolores artuum, musculorum tractum sequens; sub motu praesertim aucti; artus debiles, rigidi; it must be called rheumatism.

I am, &c.

*Nottingham, May 26, 1805.* JOHN WHITLAM.

